# Validation and application of computer vision algorithms for video-based tremor analysis

**DOI:** 10.1038/s41746-024-01153-1

**Published:** 2024-06-21

**Authors:** Maximilian U. Friedrich, Anna-Julia Roenn, Chiara Palmisano, Jane Alty, Steffen Paschen, Guenther Deuschl, Chi Wang Ip, Jens Volkmann, Muthuraman Muthuraman, Robert Peach, Martin M. Reich

**Affiliations:** 1 Center for Brain Circuit Therapeutics, Brigham and Women’s Hospital, Boston, MA USA; 2 Harvard Medical School, Boston, MA USA; 3 Department of Neurology, University Hospital Wurzburg, Wuerzburg, Germany; 4 Wicking Dementia Research and Education Centre, College of Health and Medicine, University of Tasmania, Hobart, Tasmania Australia; 5 Department of Neurology, University Kiel, Kiel, Germany; 6 Department of Brain Sciences, Imperial College, London, UK

**Keywords:** Movement disorders, Biomarkers, Neurological manifestations

## Abstract

Tremor is one of the most common neurological symptoms. Its clinical and neurobiological complexity necessitates novel approaches for granular phenotyping. Instrumented neurophysiological analyses have proven useful, but are highly resource-intensive and lack broad accessibility. In contrast, bedside scores are simple to administer, but lack the granularity to capture subtle but relevant tremor features. We utilise the open-source computer vision pose tracking algorithm Mediapipe to track hands in clinical video recordings and use the resulting time series to compute canonical tremor features. This approach is compared to marker-based 3D motion capture, wrist-worn accelerometry, clinical scoring and a second, specifically trained tremor-specific algorithm in two independent clinical cohorts. These cohorts consisted of 66 patients diagnosed with essential tremor, assessed in different task conditions and states of deep brain stimulation therapy. We find that Mediapipe-derived tremor metrics exhibit high convergent clinical validity to scores (Spearman’s *ρ* = 0.55–0.86, p≤ .01) as well as an accuracy of up to 2.60 mm (95% CI [−3.13, 8.23]) and ≤0.21 Hz (95% CI [−0.05, 0.46]) for tremor amplitude and frequency measurements, matching gold-standard equipment. Mediapipe, but not the disease-specific algorithm, was capable of analysing videos involving complex configurational changes of the hands. Moreover, it enabled the extraction of tremor features with diagnostic and prognostic relevance, a dimension which conventional tremor scores were unable to provide. Collectively, this demonstrates that current computer vision algorithms can be transformed into an accurate and highly accessible tool for video-based tremor analysis, yielding comparable results to gold standard tremor recordings.

## Introduction

Tremor syndromes are among the most common neurological disorders. Of these, essential tremor affects up to 4.6% of the global population ≥65 years old^1^. This disorder is characterised by a mixture of postural and kinetic tremors, which likely represent diverse facets of pathological oscillations in brain motor networks^2–4^. Tremor is often accompanied by additional neurological signs such as dystonia or ataxia. As such, tremor is also a common symptom in a range of acquired and genetic neurological disorders, posing a significant diagnostic challenge in clinical neurology. This translates into high rates of misdiagnosed tremor disorders^5^, which has profound therapeutic implications in particular for deep brain stimulation (DBS), a potent neural circuit therapy for tremor disorders. DBS outcomes largely hinge on accurate patient selection, which itself is influenced by accurate tremor assessment^6^. The complexity of tremor syndromes has been a roadblock to pathogenetic and diagnostic research, which culminated in a call to redefine tremor classification through quantitative phenotyping^4^.

To this end, instrumented tremor analysis offers an unbiased and detailed assessment of key tremor features, such as frequency and amplitude, which are crucial for phenotyping^7,8^, therapeutic monitoring^9,10^, differential diagnosis^11–15^ and closed-loop neuromodulation^16^. 3D motion capture methods enable comprehensive characterisation of both tremor and associated movement abnormalities (reviewed in ref. ^17^). However, the reliance on these complex and resource-intensive methods restricts their practical use, especially in routine clinical settings.

In contemporary practice, the complex phenomenology of tremor syndromes is therefore condensed into low dimensional, ordinal rating scales. These scales represent tremor items in a non-linear, logarithmic manner^18,19^ and, despite their simplicity, suffer from considerable clinimetric limitations. One of these limitations is interrater reliability, reported to be as low as 0.1 (Cohen’s kappa)^18,20–23^.

While mobile technologies, such as smartphone accelerometers, have emerged as promising tools for tremor frequency assessment^7,24–27^, they have critical limitations, such as their reliance on calibration, sensor weight and placement^8^. Additionally, they cannot readily measure associated neurological signs.

Novel computer vision (CV) methods for marker-less pose tracking have been developed for consumer applications but are increasingly adapted in movement sciences^24^^,^^28–34^. Pilot studies have shown the feasibility of CV-based measurement of neurological motor symptoms^29,33,35^ and specifically, tremor frequency^23,31,36–38^. However, CV-based measurement of tremor amplitude remains unexplored, despite it being the key kinematic determinant of patient life quality^39^.

A key challenge for pose tracking algorithms is a generalisation to clinical contexts, where medical equipment interferes with body landmark detection and disease-related alterations of movement and posture deviate from their training data^28–30^^,40^. Tools like DeepLabCut^23,29,41^ enable supervised fine-tuning of pose-tracking algorithms with task-specific data, but the training can introduce biases that lead to overfitting. Finally, consumer CV algorithms are evaluated with static metrics (i.e., Euclidean distances in single frames) that are largely unrelated to the clinical quanta of interest (e.g. frequencies, amplitudes)^28,42^. At present, there is a critical lack of rigorous validation of CV algorithms against clinical gold standard methods and application in larger neurological patient populations^29,31^.

To address these challenges, we repurpose Mediapipe, an open-source pose tracking algorithm, for comprehensive tremor analysis. We evaluate its capability to track hands in clinical standard videos of postural and kinetic tremor assessments and use the resulting time series data to compute both fundamental and advanced tremor features. We benchmark this CV framework against gold standard methods in a cohort of patients diagnosed with essential tremor. Subsequently, we apply it to an independent, retrospective dataset of unstandardised, real-world videos from two clinical sites, examining its convergent clinical validity and capability to characterise therapeutic effects of deep brain stimulation on tremor. We assess the framework’s utility to inform diagnostic and prognostic challenges in two clinical use case scenarios. Finally, we explore how different CV architectures impact the performance of tremor analysis by comparing Mediapipe to a pose-tracking algorithm specifically fine-tuned for tremor analysis.

## Results

### Validation of the computer vision framework: tremor amplitudes

To assess the CV framework’s technical and clinical validity, we first applied it to video data from a prospectively recruited cohort of patients with a diagnosis of essential tremor and treated with thalamic DBS. Ground truth values of tremor amplitudes and frequencies were determined using laboratory gold standard technologies: marker-based 3D motion capture and simultaneous wrist-mounted accelerometery.

**Fig. 1:**
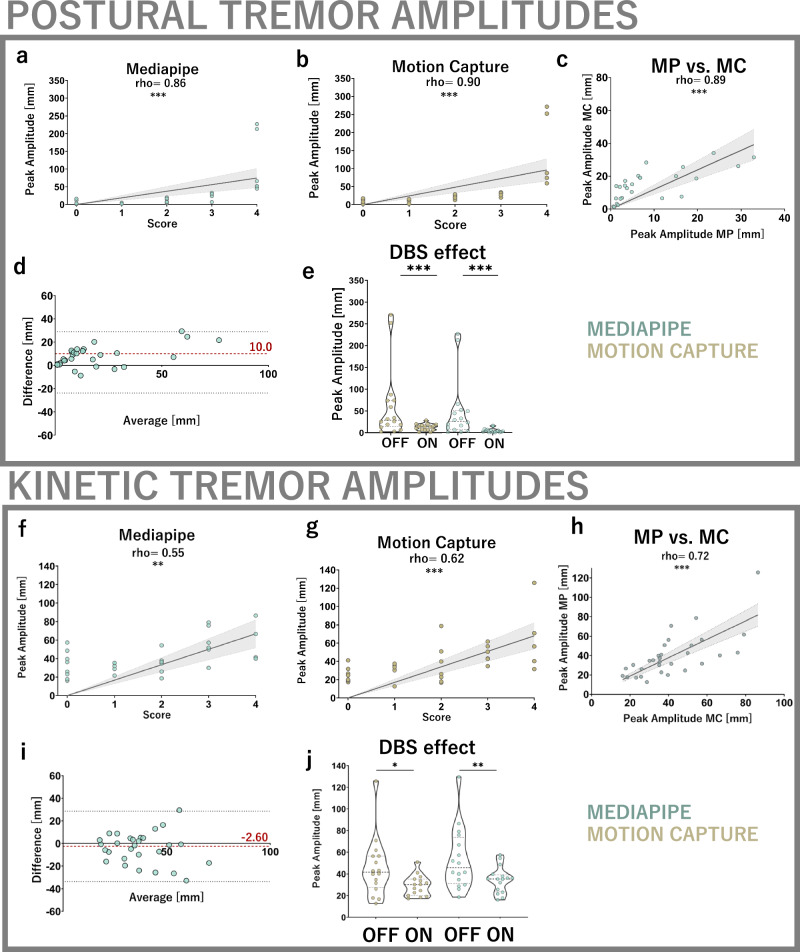
Tremor amplitude analysis. Postural. **a**–**c** Both the computer vision and motion capture outcomes show strong and significant agreement with clinical postural tremor scores assigned to each hand (MP: *ρ* = 0.86, MC: *ρ* = 0.90, both *p* < 0.001) as well as excellent inter-methodological agreement (*ρ* = 0.89, *p* < 0.001). **d** Mean deviation of computer vision-derived amplitude measurements from motion capture is 10 mm and Bland-Altman plotting shows no systematic relationship of measurement and error magnitudes. Of note, computer vision and motion capture recordings were not identical (see methods) but reflect sequential recordings. **e** Computer vision and motion capture-derived amplitudes are sensitive to DBS effect, similar to clinical scores (effect size 0.94 for motion capture, 1.00 for Mediapipe and clinical scores, all *p* < .001, Durbin-Conover corrected). Kinetic: **f**–**h** Mediapipe and motion capture-derived amplitudes show strong and significant correlations with clinical scores (MP: *ρ* = 0.55, *p* = 0.01, MC: *ρ* = 0.62, *p* < 0.001) while being strongly intercorrelated (MP vs. MC: *ρ* = 0.72, *p* < 0.001). **i** Mediapipe exhibits an accuracy for kinetic tremor tracking of −2.6 mm, as measured by the mean absolute deviation from motion capture. Bland-Altman plotting reveals no systematic relationships of error and measurement magnitudes. **j** Mediapipe and motion capture-derived amplitudes are responsive to DBS effect, with similar effect sizes (MP: 0.69, *p* = 0.003, MC: 0.60, *p* = 0.022).

CV-derived peak postural tremor amplitudes showed a strong correlation with respective clinical scores, similarly to gold standard motion capture (MP: *ρ* > 0.86, MC: *ρ* = 0.90, *p* < 0.001, Fig. 1a, b). Excellent agreement of computer vision was found with motion capture (*ρ* = 0.89, *p* < 0.001, Fig. 1c). In comparison to motion capture, computer vision had a mean absolute error of 10 mm (95% CI [5.65, 14.4]). No systematic relationship between measurement and error magnitudes was observed (Fig. 1d). Computer vision-derived tremor amplitudes fell within equivalence boundaries of motion capture tracking (±10 mm, Supplementary Fig. 1a) and were comparably responsive to DBS effect (*d* > 0.94, all *p* < 0.001, Fig. 1e), overall suggestive of equivalent accuracy. Median precision, measured by the standard deviation of each amplitude measurement, was 1.29 mm for motion capture and 0.54 mm for Mediapipe. Precision values reached equivalence to motion capture within gold-standard derived boundaries of ±3.63 mm (Supplementary Fig. 1b). Reducing the 90% CI margins to ±1.5 and ±1.0 mm did not substantially change these results, indicating robustness beyond the defined boundaries.

Mediapipe’s peak kinetic tremor amplitude estimates were strongly correlated to the clinical scores, again comparable to motion capture derived values (*ρ* = 0.55, *p* < 0.01, Fig. 1f, g). Mediapipe reached substantial agreement with motion capture (*ρ* = 0.72, *p* < 0.001, Fig. 1h). Mean absolute error was −2.60 mm (95% CI [−3.13, 8.23], Fig. 1i). Mediapipe’s accuracy in kinetic tremor amplitude measurement was equivalent to motion capture (Supplementary Fig. 1a). Mediapipe and motion capture were again comparably responsive to DBS effects on kinetic tremor amplitude (*d* = 0.69 and 0.60, Fig. 1j). Median precision of kinetic tremor amplitude measurement was calculated to be 0.31 mm for motion capture and 0.49 mm for Mediapipe. Mediapipe’s precision fell within the equivalence boundaries of ±2.1 mm (Supplementary Fig. 1c). Repeating the equivalence tests with empirically reduced 90% CI margins of ±1.5 and ±1.0 mm did not substantially change these results. Notably, the aforementioned results were similar when using mean instead of peak amplitude measurements (Supplementary Figs. 2 and 3).

### Validation of the computer vision framework: tremor frequencies

**Fig. 2:**
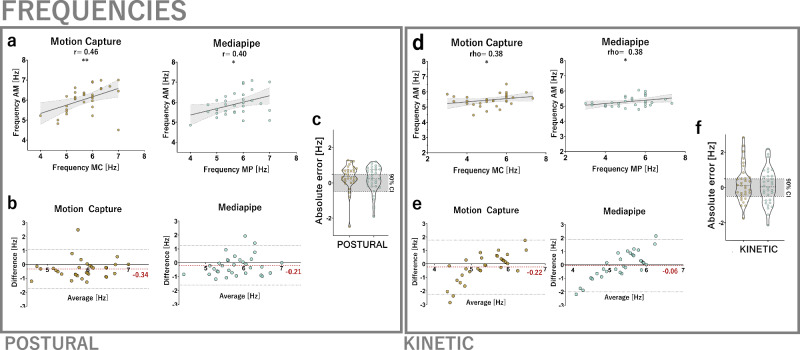
Tremor frequency analysis. **a** In the postural tremor condition, Mediapipe (MP) demonstrates strong correlations with accelerometer measurements (AM), similar to motion capture (MC), when estimating tremor frequencies (MP: *r* = 0.40, *p* < 0.05, MC: *r* = 0.46, *p* < 0.01). **b** Accuracies of postural tremor frequency measurements were calculated to be −0.34 Hz for motion capture and −0.21 Hz for Mediapipe. Bland–Altman plots revealed no systematic relationship of error and measurement magnitudes. **c** Absolute errors of postural tremor frequency measurements are equivalent to motion capture. **d** In the kinetic tremor condition, Mediapipe and motion capture-derived frequencies showed similar correlation strengths to accelerometric frequency measurements (*ρ* = 0.38, *p* < 0.05). **e** Accuracies of kinetic tremor frequency measurements were calculated to be −0.22 Hz for motion capture and −0.06 Hz for Mediapipe, however both with systematic relationships of error and measurement magnitudes. **f** Absolute errors of kinetic tremor frequency measurements are equivalent to motion capture.

Computer vision-derived tremor frequency measurements were validated against wrist-worn accelerometery, a clinical and laboratory gold standard for tremor analysis. The correspondence of tremor frequencies from Mediapipe and motion capture to accelerometery was found to be similarly strong (*r* > 0.40, Fig. 2a). The mean dominant frequency of postural tremor was measured to be 5.7 ± 0.72 Hz with accelerometery, 6.04 ± 0.65 Hz with motion capture and 5.9 ± 0.58 Hz with Mediapipe, resulting in mean absolute errors of −0.34 Hz [95% CI −0.08, 0.60] for motion capture and −0.21 Hz [95% CI −0.05, 0.46] for Mediapipe (Fig. 2b).

Within the predefined margins of ±0.5 Hz, Mediapipe-derived frequency measurements achieved equivalent accuracy to accelerometery, while motion capture exceeded the equivalence bounds (Fig. 2c and Supplementary Fig. 1d). Median precision of tremor frequency measurements was 0.58 Hz for accelerometery, 1.15 Hz for motion capture and 1.12 Hz for Mediapipe. Precision values from motion capture and Mediapipe were equivalent to accelerometer within gold standard derived margins of ±2 Hz (Supplementary Fig. 1e). Again, reducing the 90% CI margins to ±1.5 and ±1.0 Hz did not substantially alter these results.

Both motion capture and Mediapipe-derived kinetic tremor frequencies demonstrated moderate agreement with respective accelerometric measurements (motion capture: *ρ* = 0.38, *p* = 0.034; Mediapipe: *ρ* = 0.37, *p* = 0.033, Fig. 2d). The mean dominant frequency of kinetic tremor was 5.25 ± 1.06 Hz using accelerometery, 5.48 ± 0.41 Hz using motion capture and 5.31 ± 0.34 Hz using Mediapipe, with mean absolute errors of 0.22 Hz (95% CI [−0.15, 0.59]) for motion capture and 0.06 Hz (95% CI [−0.30, 0.41]) for Mediapipe. Bland-Altman plots for motion capture and Mediapipe suggested a systematic relationship between error and measurement magnitudes (Fig. 2e).

Within the predefined boundaries of ±0.5 Hz, Mediapipe’s accuracy in frequency measurements was equivalent to accelerometery (Fig. 2f and Supplementary Fig. 1d). In contrast, motion capture’s accuracy was significantly lower than Mediapipe (*T*(31) = 2.98, 95% CI of difference [0.05, 0.28], *p* = 0.006). Median precision was 0.58 Hz for accelerometry, 0.14 Hz for motion capture and 0.1 Hz for Mediapipe. Both motion capture and Mediapipe precision values fell within equivalence boundaries derived from the minimal precision achieved by accelerometery, ±2 Hz (Supplementary Fig. 1f). Reducing the margins to ±1.5 and ±1.25 Hz in equivalence tests did not substantially alter these results.

### Retrospective application: postural tremor

**Fig. 3:**
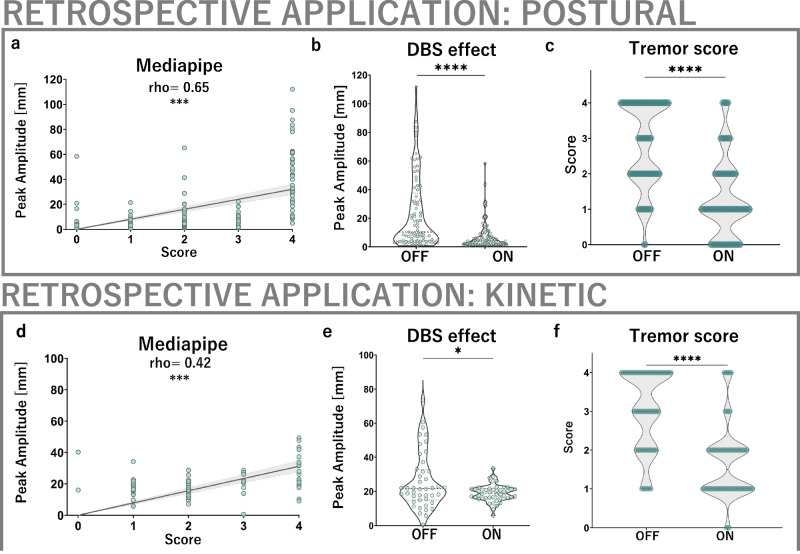
Application of computer vision tremor analysis in an independent, retrospective cohort. **a**, **b** Computer vision-derived postural tremor amplitude measurements are strongly correlated to clinical scores (MP: *ρ* = 0.65, *p* < 0.001) as well as responsive to DBS effects (MP: *r* = 0.49 (95% CI [0.34, 0.61]), *p* < 0.001; score: *r* = 0.61 (95% CI [0.49, 0.71], *p* < 0.001, **b**, **c**). **d** For kinetic tremor, Mediapipe-derived amplitude measurements are substantially correlated to respective clinical scores (*ρ* = 0.42, *p* < 0.001) and responsive to the effect of DBS (MP: *r* = 0.37, 95% CI [0.03, 0.61], *p* = 0.025; score: *r* = 0.64, 95% CI [0.48, 0.75], *p* < 0.001, **e**, **f**).

In order to clinically validate the CV framework in an independent sample, we applied it to clinical videos of 43 individuals undergoing clinical tremor assessment before and after thalamic DBS implantation. Peak postural tremor amplitudes derived from Mediapipe were strongly correlated with the corresponding tremor scores (Fig. 3a). Wilcoxon testing further revealed that the CV framework’s peak amplitude measurements were highly responsive to the effect of DBS, as were scores (Fig. 3b, c). Repeating the analyses using mean instead of peak tremor amplitudes yielded similar results with respect to score correlation (Supplementary Fig. 4). Mean dominant frequency of postural tremor was calculated to be 5.96 ± 0.76 Hz.

### Retrospective application: kinetic tremor

In the 25 available individuals, a moderate correlation was found between the measured peak amplitudes and the corresponding tremor scores (Fig. 3d). Wilcoxon testing revealed that peak kinetic tremor amplitude measurements were highly sensitive to the DBS effect (Fig. 3e, f). Repeating the analyses using mean instead of peak amplitudes yielded similar results (Supplementary Fig. 3). The mean dominant frequency of kinetic tremor was calculated to be 5.75 ± 0.58 Hz.

### Clinical use cases: diagnostic features

Instrumented tremor analysis can provide valuable differential diagnostic clues for tremor syndromes. Beyond the basic tremor characteristics like amplitude and frequency, advanced features such as harmonics or inter-limb tremor coherence have previously been established to support differential diagnosis of tremor syndromes^10^. To this end, we investigated whether the CV framework is capable of extracting advanced diagnostic tremor features, which usually require electromyography or other sensors.

**Fig. 4:**
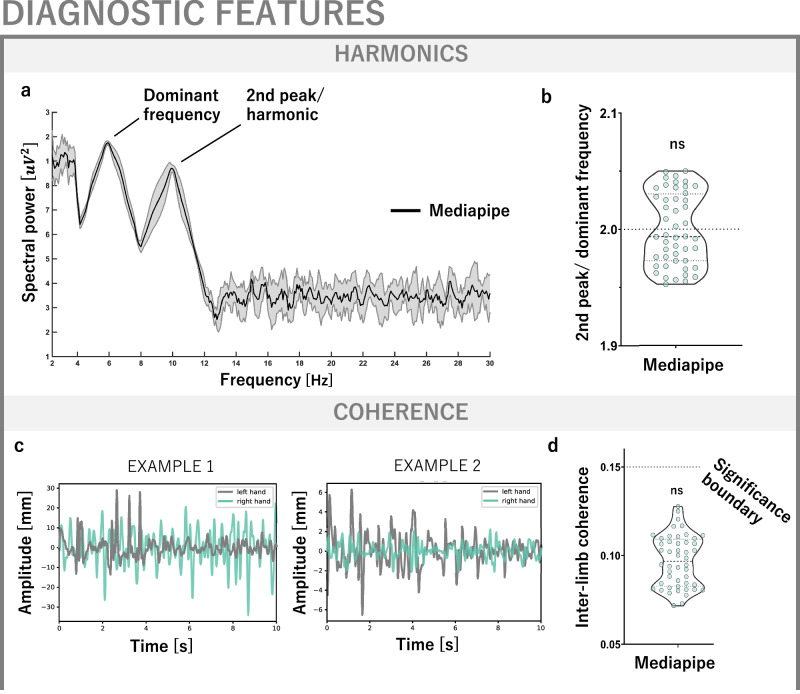
Using the CV framework to augment diagnostic insight. **a** Example power spectrum derived with Mediapipe. In addition to the primary, dominant frequency peak, there is a clear cut second peak, i.e. a harmonic. **b** In line with previous electromyographic work^43^ demonstrating the diagnostic value of the presence of a harmonic at twice the dominant tremor frequency, we divided the frequencies of the second and first frequency peak and performed a one-sample t-test against 2.0, which was not significant (*p* = 0.99, SD of discrepancy 0.03). **c** Mediapipe-derived tremor signals from both hands are not coherent. **d** On a group level, interlimb coherence values fall below the time series length-dependent significance value of 0.15 (median MP: median 0.097, maximum 0.13), in line with electromyographic literature^43^.

Indeed, the Mediapipe-derived tremor signal displayed a harmonic peak which was located at twice the mean dominant frequency (Fig. 4a, b), a feature previously reported to differentiate essential from parkinsonian tremor^43^. Moreover, no significant inter-limb tremor coherence was detected, a feature reported to discern essential tremor from orthostatic tremor^10,44^ (Fig. 4c, d).

### Clinical use cases: predictive modelling

Albeit efficacious in the majority of cases, thalamic DBS outcomes vary^9^. Lack of tremor improvement or even paradoxical increases in kinetic tremor amplitude signify a poor DBS outcome^6^. Patient-specific factors such as baseline clinical tremor scores have been shown to aid DBS outcome prognostication across tremor disorders^45^, which facilitates patient counselling.

**Fig. 5:**
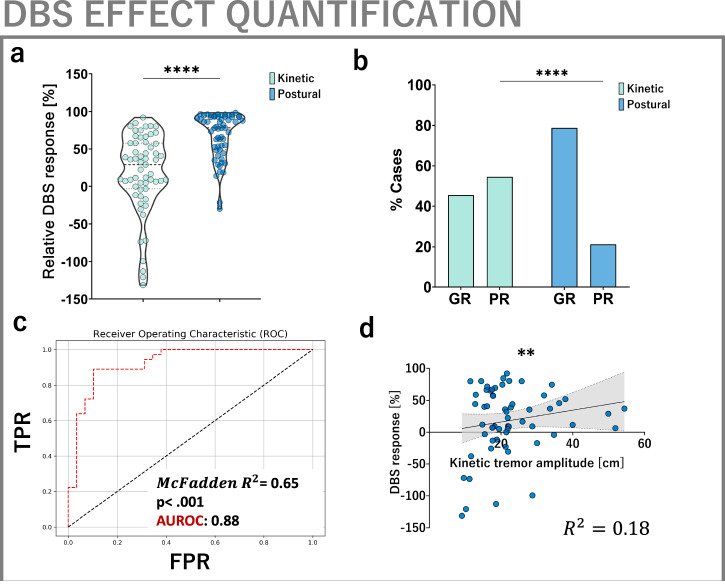
Using the CV framework for DBS effect quantification and prognostication. **a** DBS exerts stronger effects on postural than kinetic tremor as measured by relative amplitude reduction (*p* < 0.001, effect size = 0.72). **b** Binarizing the sample into good (GR) and poor responders (PR) using a combined criterion of ≥2 cm tremor amplitude and ≤30% amplitude decrease in DBS ON yields a significant greater fraction of poor responders in the context of kinetic tremor, which is an important driver of functional disability (55% vs. 21% of cases, *p* < 0.001). **c** Preoperative hand kinematic features are strong and significant predictors of DBS outcome (*χ*^2^ = 58.4, *p* < 0.001, McFadden *R*^2^ = 0.65). Baseline kinetic tremor amplitude is found to be a significant and independent predictor of DBS outcome for kinetic tremor. Leave-one-out-cross-validation evaluation yields an AUROC of 0.88, a balanced accuracy of 0.88, F1-score 0.89. **d** Moreover, baseline kinetic tremor amplitude is a significant predictor of the relative DBS response in a linear model (*p* = 0.012), as measured by the percentual reduction of kinetic tremor amplitude (*R*^2^ = 0.18, Pearson’s *r* = 0.35, *p* = 0.005).

Therefore, we aimed to assess the utility of computer vision-derived metrics in predicting DBS outcomes from preoperative kinematics and clinical score information. First, we found that kinetic tremor was markedly less strongly modulated by DBS than postural tremor (Fig. 5a). Since persisting kinetic tremor is a key driver of functional disability in essential tremor and among the main reasons for failed DBS interventions^46,47^, we binarized our patient cohort into good and poor responders based on post-operative tremor amplitudes. We chose a threshold of ≥2 cm residual tremor amplitude and ≤30% relative tremor reduction in DBS ON, so as to identify cases with clinically relevant disability^6,46,47^. Applying this threshold, we found that kinetic tremor was significantly more frequently associated with a poor outcome (55% vs. 21% fraction of poor responders, *p* < 0.001, Fig. 5b).

To identify determinants of suboptimal DBS outcomes, which might assist in preoperative patient counselling, we conducted a logistic regression analysis. Using binarized response group as the outcome variable and preoperative limb kinematic features as covariates, we detected a strong and significant association of preoperative tremor measurements to DBS outcomes (*χ*^2^ = 58.4, *p* < 0.001, McFadden *R*^2^ = 0.65). Among all covariates, baseline kinetic tremor amplitude emerged as a significant and independent predictor of DBS response (*p* = 0.002, OR 0.89, 95% CI [0.82, 0.96]). Implementing a rigorous leave-one-out cross-validation to evaluate the model’s performance yielded an area under the receiver operator curve of 0.88 and a F1-score of 0.89 (Fig. 5c). Moreover, baseline kinetic tremor amplitude emerged as an independent predictor of DBS-associated improvement of kinetic tremor amplitude in a linear regression model (*R*^2^ = 0.18, *p* < 0.001; baseline kinetic tremor: *p* = 0.021, Fig. 5d). Of note, preoperative tremor scores were neither a significant predictor of binary outcome nor tremor amplitude change. For additional exploration of clinical and demographic features across cohorts, please see supplementary results.

### Assessment of a disease-specific convolutional network: DLC-RCNN

The performance of pose tracking algorithms is crucially influenced by task and visual context, especially in clinical settings^28,29,35^. To gauge this effect’s relevance in the context of tremor, we additionally developed a tremor-specific residual convolutional neural network using DeepLabCut^41^ (DLC-RCNN). This network was trained with >120,000 frames of clinical video material. Final performance evaluation showed a median Euclidean distance of 3.56 mm and 10.74 mm between user-annotated and predicted keypoints, demonstrating acceptable generalisation and tracking accuracy related to fingertip size (occupying 10-20 pixels, corresponding to 10–20 mm on average^29,48^). The model’s generalisation to an out-of-sample validation dataset (>15,000 frames) showed high confidence in predicting postural tremor keypoints (median likelihood of 0.99, 4884 predictions) but unacceptably low confidence for kinetic tremor keypoints (median likelihood of 0.22, 10,532 predictions, Supplementary Fig. 5). Therefore, DLC-RCNN could only be used for postural tremor analysis.

**Fig. 6:**
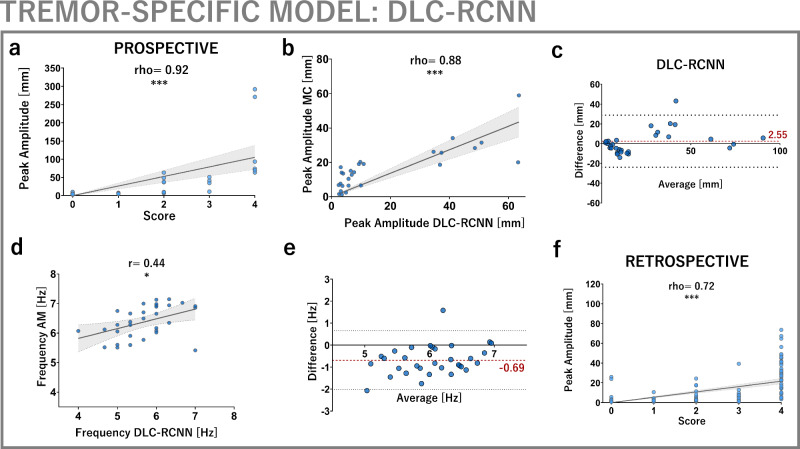
Application of a disease-specific convolutional neural network for postural tremor analysis across cohorts. **a**–**c** In the prospective cohort, DLC-RCNN-derived amplitude measurements are strongly correlated to clinical scores (*ρ* = 0.72, *p* < 0.001) and motion capture (*ρ* = 0.88, *p* < 0.001). Mean absolute error is 2.55 mm (95% CI [−2.11, 7.29]) with no systematic relationship to measurement magnitudes. **d**, **e** DLC-RCNN frequency measurements are moderately correlated to accelerometer (*r* = 0.44, *p* < 0.05) with a mean absolute error of −0.69 Hz [95% CI −0.93, 0.44]. **f** In the retrospective cohort, DLC-RCNN-derived postural tremor amplitudes show a moderate correlation to clinical scores (*ρ* = 0.72, *p* < 0.001). DLC-RCNN however failed to capture and measure kinetic tremor in both cohorts.

In the prospective cohort, DLC-RCNN-derived tremor amplitudes were strongly correlated to clinical scores (*ρ* = 0.92, *p* < 0.001) and gold standard motion capture (*ρ* = 0.88, *p* < 0.001, Fig. 6a, b). The mean absolute error was 2.55 mm (Fig. 6c). DLC-RCNN-derived mean dominant tremor frequencies were moderately correlated to accelerometer (*ρ* = 0.44, *p* < 0.05), with a mean absolute error of −0.69 Hz (Fig. 6d, e). In the retrospective cohort, DLC-RCNN-derived postural tremor amplitudes were moderately to strongly correlated with assigned clinical scores (*ρ* = 0.72, *p* = 0.001, Fig. 6f). DLC-RCNN’s accuracy and precision (0.66 mm) for amplitudes was equivalent to motion capture. The mean dominant frequency was calculated to be 6.38 ± 0.54 Hz, but the DLC-RCNN’s frequency accuracy was significantly lower than Mediapipe and motion capture, hence not equivalent.

## Discussion

Tremor disorders underscore the critical need for granular phenotyping in clinical management. While traditional instrumented methods provide valuable insights, their high resource demands significantly limit their widespread application in clinical settings. As a result, clinicians often rely on a more reductionist approach, employing semi-quantitative rating scales that bear considerable clinimetric limitations^18,20,21^. Our study was aimed to address these challenges by comprehensively assessing the feasibility and robustness of computer vision methods enabling tremor analysis from standard clinical videos.

First, we found that the CV framework achieves comparable accuracy to specialised gold standard equipment in the measurement of both tremor amplitude and frequency. Second, we demonstrated its practical utility not only in characterising the effects of deep brain stimulation but also in providing valuable insights into diagnostic and prognostic challenges – aspects that conventional scores failed to capture. Finally, our study elucidated the impact of different algorithmic architectures on clinical pose tracking capability, providing a roadmap for future technical scalability.

The results of our prospective validation underline the framework’s accuracy, precision and clinical validity, which largely match gold standard equipment. While prior studies have tapped into the potential of computer vision for tremor detection^38,49^ and frequency extraction^24^, amplitude quantification remained largely unexplored. Yet, tremor amplitude is pivotal in assessing patient disability and therapeutic outcomes^6,9^. Our findings indicate that smartphone videos, coupled with computer vision tracking tools, can gauge tremor amplitude with an accuracy of up to 2.6 mm, a value that falls on the low end of reported pose tracking accuracies^28,32^ and that is almost an order of magnitude smaller than the lowest anchor value provided in the tremor rating scale (20 mm).

Compared to gold standard accelerometery, computer vision-derived tremor frequency measurements demonstrated a mean absolute error between −0.06 and −0.21 Hz, values falling well within, if not below modern vision-based frameworks^24^. More generally, large scale studies investigating clinical pose tracking in other movement disorders^33,35^ report moderate to high score correlation strengths in the range of 0.6–0.8, which corresponds closely to our reported values of 0.55–0.86. Overall, this is strongly indicative that the CV framework effectively captured the clinically relevant target information.

Notably, some correlation plots exhibit increasing residuals with higher scores, which is well in line with the notion of a logarithmic rather than linear relationship of tremor severity and ordinal scores^18^. Continuous digital biomarkers are not subject to such non-linearity, which often complicates both intra- and interindividual comparisons relevant for clinical studies and management.

Therefore, CV frameworks offer the potential to dramatically simplify tremor analysis by eliminating the need for multiple devices and sensors and even enabling the analysis of unstandardised legacy videos, underscoring their generalisability and versatility. The fully vision-based approach can be further scaled to additionally quantify tremor-associated neurological signs such as ataxia^50^ or dystonia^35^. This capability aligns with the central goals of future quantitative phenotyping efforts in tremor disorders^4^.

Next, we applied the CV framework in exemplary use cases that are directly inspired by clinical tremor management. Mediapipe was capable of extracting advanced diagnostic tremor features, which offer additional insights relevant for the differential diagnosis of tremor disorders^10,43,44,51^. For example, a harmonic peak at twice the dominant tremor frequency or a lack of inter-limb tremor coherence can be diagnostic clues differentiating essential tremor from other tremor syndromes^10,43,44^. While our study was not designed to facilitate comparisons across different tremor disorders, our results nonetheless demonstrate the feasibility of using the CV framework to derive diagnostically relevant tremor features, linking computer vision-derived biomarkers with sensor- or EMG-based findings reported in the neurophysiological literature^10,43,44^.

Second, CV-derived features could aid in characterising thalamic neurostimulation outcomes. Our predictive model, focusing on kinetic tremor reduction as the key determinant of disability and life quality after DBS implantation^3,6,9,46,52^, identified baseline kinetic tremor amplitude as a predictor of DBS outcome. Interestingly, conventional tremor scores lacked this predictive power, emphasising the advantages of sensitive and continuously encoded digital biomarkers in capturing such nuanced clinical relationships. This finding aligns with similar results for DBS outcome prediction based on scores in Parkinson’s disease^45^ as well as emerging evidence for the added value of digital phenotyping in neurological disorders which reaches far beyond conventional scores^29,33,35,53,54^.

While both CV architectures excelled at postural tremor tracking, their performance was reduced in kinetic tremor tracking. The tremor-specific DeepLabCut model entirely failed to track kinetic tremor, drastically reducing its versatility. We hypothesise that Mediapipe outperformed the disease-specific model due to its 3D pose tracking capability and high hand landmark coverage (21 landmarks), which is essential for tracking the complex configurational changes of the hands during the finger-to-nose test^28,55,56^. Similar observations were reported for head tracking in the context of dystonia^35^, while another study found that a disease-specific network trained with DeepLabCut outperformed Mediapipe in the tracking of abnormal eye movements^29^. As the interactions of task, context and algorithm selection for clinical pose tracking are just beginning to be unravelled^28,55^, future research is needed to explore the benefits of 3D tracking capabilities, task-specific algorithm customisation and model combination in different clinical scenarios.

Several limitations should be acknowledged. While effective across task conditions, Mediapipe hands was less robust during kinetic tremor assessments involving complex hand configurations. In addition, it does not track proximal arm landmarks, which could be of interest for future mechanistic investigations of DBS effects^57,58^. In future clinical pose tracking studies, a combination of algorithms that synergise 3D and full-body tracking with task-specific customisation could offer a more comprehensive approach to tremor analysis across different body regions. Second, the CV framework was highly accurate, but our sequential recording strategy – adopted to minimise marker interference which could lead to overly optimistic tracking results – might introduce biological variance in tremor amplitude measurements^59^. This approach is likely to underestimate rather than overestimate the framework’s accuracy, indicating that the technical agreement between the CV framework and gold standard might, in fact, be even higher. Lastly, while the CV framework was effective across both cohorts, the prospective cohort was limited in size and recruited from a single centre. In the future, larger multi-centric studies are needed to confirm the broader applicability and robustness of CV-based tremor analysis in varied clinical settings.

In conclusion, repurposing open-source pose tracking algorithms like Mediapipe enables tremor analysis from standard clinical video material with comparable accuracy to gold-standard methods and high convergent clinical validity. This approach enables the extraction of digital biomarkers for tremor diagnosis and prognosis and represents a rapidly scalable alternative to more resource-intensive and marker-based methods. Future work should focus on exploring hybrid approaches, combining different pose-tracking algorithms for a more comprehensive analysis of tremor across body regions and task conditions. We envision computer vision pose tracking as a pivotal tool to strengthen and democratise digital and precision medicine approaches in Neurology.

## Methods

### Ethics approval

This study was conducted in accordance with the Declaration of Helsinki and ethics approval was obtained from the Julius-Maximilians University Wuerzburg’s ethics committee (#283/14 and 163/14_MP). Patients provided written informed consent for all experimental procedures.

### Study cohorts and design

**Fig. 7:**
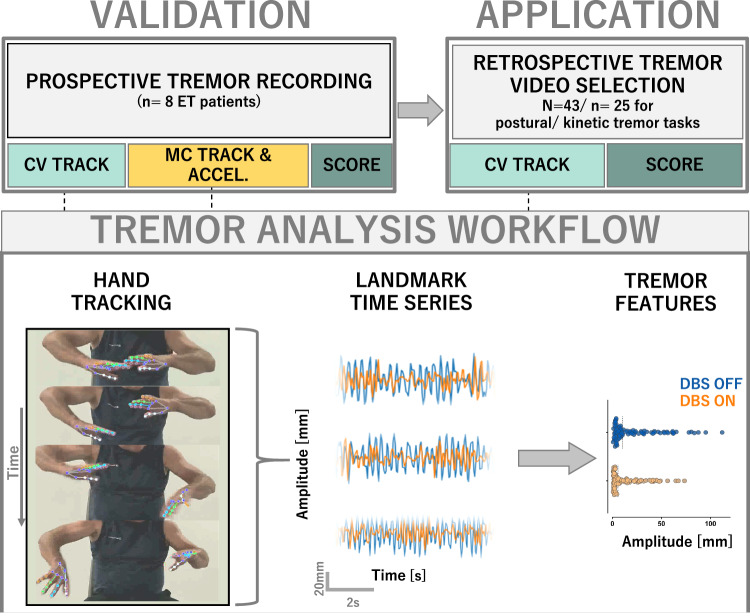
Workflow. The study was conducted into two independent cohorts/phases. In the prospective validation phase, upper limb tremor was recorded with a state-of-the-art motion capture (MC) and wrist-worn accelerometer (ACCEL) setup, as well as with a smartphone camera for subsequent computer vision-based analysis (CV). Additionally, tremor was scored using the Fahn-Tolosa-Marin clinical tremor rating scale for each limb. Each hand’s time series data extracted from motion capture, accelerometer and computer vision analysis was passed into a common analytical pipeline to compute canonical tremor features for clinical and inter-methodological correlation. Finally, the computer vision-based tremor analysis was applied to a retrospective video dataset of ET patients before and under chronic DBS treatment for additional clinical validation in an independent dataset. Given the largely unstandardised video recordings, video selection criteria were applied in order to ensure sufficient data quality, in accordance with best practice considerations in computer vision movement analysis. Subjects were included for tremor analysis if: (i) at least one preoperative as well as one postoperative follow-up video under active DBS was available, (ii) video material was free of excessive camera movements, (iii) video material did not contain visual distortion such as different zoom depths, (iv) tracked limbs were fully visible in the video frame throughout the recording, and (v) video material contained at least 5 seconds of continuous arm holding for postural and 3 repetitions of finger-to-nose pointing per side for kinetic tremor assessment. Informed consent to disclose images was obtained from the patient shown.

The study consists of two independent phases with independent cohorts. This design was chosen to reflect best practices in machine learning, aiming to ensure validity, generalisability and reproducibility (Fig. 7). All patients had a diagnosis of essential tremor based on the Movement Disorder Society’s consensus criteria^4^ and active bilateral thalamic deep brain stimulation, programmed to individually optimal settings. All patients were refractory to anti-tremor medication (propranolol, primidone).

The retrospective cohort consisted of *n* = 58 patients (mean age at surgery 66.4 ± 9.84 years, 32 males, mean disease duration at surgery 30.4 ± 19.3 years). 14 patients underwent DBS surgery at Wuerzburg University Hospital between 2016 and 2017 and the remaining 44 patients at Kiel University Hospital between 2003 and 2015^13,60,61^. The mean postoperative interval to videotaping was 25.8 ± 20.8 months. Participants were video recorded whilst seated in a clinic room with standard ambient lighting. All videos were collected using various consumer grade, handheld or tripod mounted cameras in the context of standard clinical care to document the severity of postural and kinetic tremor components. Spatiotemporal video resolution was at least 25 Hz and 320 ×238 pixels (px), respectively. The Fahn-Tolosa-Marin Tremor Rating Scale (FTM) was formally administered before DBS surgery and again during optimal DBS settings, by movement disorders specialists^13,60–62^. Separate video segments showing the assessment of postural tremor (arms in front of chest, “wing-beating position”, fingertips facing each other but not touching) and kinetic tremor (minimum of three repetitions of finger-to-nose-test on both sides) were identified. Clinical score distributions are shown in Supplementary Fig. 4a.

In the retrospective cohort, video quality criteria were utilised to optimally balance standardisation, robustness and broad applicability. Videos were excluded for computer vision analysis if the hands left the frame and if excessive camera movements or zooming were present. These criteria reflect a practical consensus synthesised from previous work in computer vision for movement analysis^24,28,29,30^ as well as exploratory pilot experiments preceding this study. Based on these criteria, *n* = 15 patients had to be excluded for postural and *n* = 33 patients for kinetic tremor tracking, leaving videos of *n* = 43 and *n* = 25 individuals for subsequent computer vision-based hand tracking in postural and kinetic conditions, respectively.

The prospective cohort consisted of *n* = 8 patients (mean age at surgery 66.6 ± 10.4 years, 4 males, mean disease duration at surgery 30.2 ± 19.5). All patients were recruited from the movement disorders clinics at the University Hospital Wuerzburg, department of Neurology, in 2021. The mean postoperative interval to the experiment was 53.6 ± 33.3 months.

### Prospective experimental design

Experiments were conducted at the department of Neurology, University Hospital Wuerzburg. Participants underwent standardised assessment of postural (holding arms in front of chest, “wing-beating position”, fingertips facing each other but not touching, 3 blocks of 30 seconds) and kinetic tremor (15 repetitions of finger-to-nose pointing per side, each starting from a resting position of the laterally outstretched arm). Tremor assessments were recorded in a 2 × 2 block design with DBS (on/off) and method (video/motion capture and accelerometery) as intraindividual factors. Minimal DBS washout period after impulse generator deactivation was conservatively set to 45 minutes to exclude stimulation carry over effects^63,64^. Experimental blocks were pseudorandomized to reduce systematic biases. Based on the video material, the corresponding items of the FTM tremor rating scale (postural and kinetic tremor amplitudes) were annotated by a clinician expert in movement disorders blinded to the experimental condition (MMR).

### Motion capture and accelerometery setup

A six-camera optoelectronic motion capture system (SMART-DX, BTS, Italy) operating at a temporal resolution of 100 Hz was used to track retroreflective markers placed bilaterally on the upper limbs’ ulnar styloid, lateral epicondyle of the humerus and the acromion, as previously described^14^. Two additional markers were placed on the middle and index fingertips’ dorsal heads for the assessment of postural and kinetic tremor, respectively. The signals for postural and kinetic tremors were computed from the middle and index fingertips’ signals and exported for subsequent computation of tremor characteristics. During all recording sessions, two inertial measurement units (Opal, APDM, USA; dimensions: 48.5 × 36.5 × 13.5 mm; size: 22 g) were placed on the dorsum of both wrists. Tri-axial accelerometer data were used to measure tremor frequencies (sampling frequency: 128 Hz). To avoid potential interference of retroreflective markers with computer vision tracking, experiments were repeated without markers for sequential analysis, as previously described^29^.

### Video hand tracking setup

Participants were seated on a chair in front of a neutral background. Tremor assessment was videotaped using a standard smartphone camera (Samsung Galaxy S20, Samsung, Seoul, South Korea), operating at a spatiotemporal resolution of 1920×1080 px and 60 Hz. The camera was mounted on a standard tripod in landscape mode at a viewing distance of 3 metres to cover the full body of the participants centrally in the video frame throughout the recording time. To avoid obscuring anatomical landmarks, participants were asked to wear sleeveless tops exposing the shoulders and arms. Watches or other jewellery were removed or covered with tape to prevent any interference with the limb tracking, e.g., through aberrant reflections. For videos, pixel-to-metric conversion was derived using a “ChArUco” board (a checkerboard with additional geometric shapes of known metric dimensions for calibration), which was presented before each new video run, as previously described^29^. Motion capture markers and accelerometers significantly change the visual appearance of hands, which impacts computer vision tracking performance and reduces external validity in non-instrumented settings. Hence, motion capture combined with accelerometery and computer vision recordings were taken separately.

### Mediapipe

For video-based hand tracking, we utilised a powerful and widely used computer vision and pose tracking framework, Mediapipe^31,65^ (MP). To this end, the Mediapipe PyPI package was executed in Python Version 3.9 and the respective hand landmark detection model applied to the video dataset which loaded using OpenCV^66^. Based on Mediapipe’s internal computation of “world referenced landmarks”, no further calibration step was needed and the coordinate time series of the 21 landmarks per hand were exported for subsequent calculation of tremor characteristics.

### Tremor-specific convolutional neural network

Additionally, a residual convolutional neural network was fine-tuned using DeepLabCut^41,48^ to track 29 upper body landmarks from diverse clinical videos (henceforth DLC-RCNN). In an iterative process, frames were extracted from a total of 202 clinical videos from 58 retrospective patients as well as 10 videos from 10 healthy controls performing the finger-to-nose test. These videos were deliberately taken in diverse video settings (perspective, lighting, background) to model variance typical to medical videography^29,67^. To further broaden the coverage of variability in regards to anthropomorphic, pose, lighting, background and other technical factors^29^, a *k-Means* algorithm was utilised for frame extraction from videos. Extracted frames were subsequently labelled by a trained annotator (AJR) and validated by an expert annotator (MF). In order to minimise labelling errors interfering with training efficiency, the labelled frames were plotted and checked for accuracy and plausibility before the annotated frame sets were passed into neural network training using 95% of data, leaving the remainder as a test set for performance evaluation. In a total of 13 consecutive iterations, the CNN was initialised with ResNet-50 weights and trained using both *default* and *imgaug* augmentation approaches. To ensure sufficient convergence of the loss function, the maximum iterations were varied between 500,000 and 1,030,000.

In addition, a subset of 10 retrospective clinical videos was held back for an additional out-of-sample validation. Importantly, no videos of the prospective cohort were included in CNN training in order to maintain strict separation between training, test and validation datasets across the study arms. Model performance was evaluated in a multi-faceted approach as previously described^29,68^ (Supplementary Fig. 5).

### Calculation of tremor characteristics

As previously reported^29^, an inverse relationship of the DLC-RCNN’s tracking performance and spatial resolution of the videos was observed. Therefore, videos were resampled to 1280×720 px. This value offered the optimal trade-off of spatial information for landmark tracking and favourable tracking performance.

Kinematic analysis of limb movements was implemented in Python 3.9 using standard scientific analysis packages (pandas, sklearn, numpy, scipy) and a custom analysis pipeline. Two-dimensional coordinate time series were conditioned by removing low likelihood marker data points (confidence/likelihood <0.5, default setting in Mediapipe). Mediapipe outputs three-dimensional marker coordinates, but for fair comparison to the two-dimensional DLC-RCNN, only the x and y coordinates were used. The missing points were then interpolated using a linear filter. Furthermore, a high pass filter was implemented to remove remaining low-frequency components associated with slow arm drifts unrelated to tremor frequency. Given its consistency at successful tracking across videos and its clinical relevance we tracked the middle finger’s distal phalanx for postural tremor analysis (Mediapipe marker “middle finger tip”) and tracked the index finger’s distal phalanx (Mediapipe marker “index finger tip”) for kinetic tremor quantification. A bandpass filter was implemented (postural: low cut = 1 Hz, high cut = 10 Hz), removing both high frequency noise (such as “prediction jitter” introduced by frame-to-frame tracking variability, or failure) and low-frequency large-scale movements (such as slow arm drifts unrelated to tremor frequency). To correct for occasional poor tracking, an additional spiking threshold was applied that identified markers that differed from the previous marker coordinates by over 100px and removed them prior to linear interpolation (this was only relevant in a minority of kinetic tremor videos).

For pixel-to-metric conversion, an individual scaling factor was calculated for each retrospective clinical video in which no systematic calibration information (i.e., checkerboard) was available. To this end, the interpupillary distance (IPD, pupil centre to pupil centre) was used, as previously described^29^. The ground truth metric IPD was derived from each individual’s preoperative structural T1w-MRI scans (averaged over three measurements) using Suretune 3 (Medtronic Inc., Minneapolis, MN, USA). A patient-specific scaling factor was then calculated by the real IPD (in mm) divided by the video IPD (in px) measured with the open-source software GIMP 2.8.22 (GNU Image Manipulation Program). In the prospective videos, a ChArUco board was used for pixel-to-metric conversion. Using these scaling factors and the known temporal video resolution, the time series could be spatiotemporally transformed to facilitate meaningful comparisons with clinical tremor scores, which mainly rely on tremor amplitude estimates, as well as previous tremor research.

Tremor amplitude was calculated by first computing a spectrogram of the tremor signal which describes the power density of frequencies of a signal as it varies with time. The frequency bin with the maximum power is identified for each frame and the associated amplitude and frequency for that frame is stored. Finally, the resulting feature time series were collapsed per experimental condition (i.e., task, DBS status) into their aggregated mean and peak values. In accordance with the clinical scoring assessing maximal amplitude, peak values were primarily used for subsequent comparisons and correlations. Of note, all main results were reproducible using mean instead of peak amplitudes (Supplementary Figs. 3 and 4).

### Computation of tremor frequency from wrist-worn accelerometer

Firstly, the axis with the highest range of variation in acceleration was identified for further analysis. The data underwent pre-processing, involving bandpass filtering between 1 and 10 Hz using a 5th order Butterworth filter. The trials were then segmented based on the type of tremor. Postural tremor trials were segmented by means of synchronised video recordings (VIXTA, BTS, Italy), by excluding any initial or final voluntary arm movements. Kinetic tremor trials were segmented by thresholding the moving average of the absolute value of the signal. For both types of tremor, power spectral density (PSD) of the accelerometric signal was calculated using the *pwelch* method with a rectangular window of 1 s duration and a 0.5 s overlap. Frequency peaks in the PSD were identified, and average values and standard deviations across trials were calculated for each patient and condition.

### Computation of advanced diagnostic features from motion capture and computer vision

To further characterise the nature of the tremor, we analysed harmonics based on the spectrograms derived from each method. Beyond the dominant frequency, we identified additional peaks, indicative of potential harmonics. To determine the nature of these harmonics, we focused on the second most prominent peak in the spectrogram. The frequency of this second peak was divided by the dominant frequency to determine the harmonic relationship. A resulting value of 2 would indicate that the second peak is an even-numbered harmonic, which has previously been reported to be differential diagnostically relevant^43^. Also, we analysed the inter-limb coherence between the tremor signals of both hands. Inter-limb coherence provides a measure of the synchrony or similarity between the tremor oscillations in the two hands. For essential tremor, a diagnostic feature is the presence of non-coherent tremors between the two hands, as previously described^44^. To quantitatively determine the coherence, we calculated the coherence value between the tremor signals of the two hands, derived from both motion capture and computer vision. Based on the length of the time series data, a significance threshold of 0.15 was established. Coherence values below this threshold were considered as non-coherent, consistent with essential tremor, while values above this threshold suggested coherent tremor activity between the hands.

### Statistical methods

For the definition of equivalence boundaries, the smallest effect size of interest (SESOI)^69,70^ was chosen in accordance to the anchor intervals used in the FTM tremor scale. We reasoned that the minimal clinically relevant difference of tremor amplitudes most likely corresponds to the boundary between score 0, “no tremor” and score 1, “slight tremor”. However, no amplitude estimate is used as an anchor for this differentiation in the Tremor scale. Based on the 20 mm intervals used to differentiate the subsequent score levels 2–4, which align with our clinical experience in terms of tremor relevance to everyday life, as well as previous technical validations of sensor-based tremor amplitude measurements achieving accuracies ±10 mm^71^, a SESOI of 10 mm was empirically derived. Half of the Tremor score anchor interval, this value was intended to reflect a rather conservative estimate of the minimally clinical important difference. For tremor frequency, a SESOI of 0.5 Hz was chosen in accordance with similar previous work^24^. For precision metrics, no a priori information was available. In line with previous work^29^, the respective SESOI was anchored at the minimum precision calculated from gold standard, accelerometry. To exclude significant confounding by potential outliers, equivalence boundaries were empirically lowered to determine a hypothetical minimum, given the data. Equivalence was assessed with the two one-sample t-test (TOST) method as implemented in JAMOVI Version 2.2.5.0.

Leave-one-out cross-validation (LOOCV) was used to evaluate the performance of the logistic regression model for DBS outcome prediction. We used kinematic features and the binarized outcome variable based on a ≥ 30% kinetic tremor amplitude increase for analysis. The LOOCV procedure involved iteratively training the model on all samples except one, which was then used as the test set. This process was repeated for each sample, resulting in multiple rounds of training and testing. Performance metrics, such as accuracy, precision, recall, F1-score, AUROC, balanced accuracy and the confusion matrix, were calculated to assess the model’s predictive capability. Additionally, we performed a Wald test to determine the significance of each selected feature as an independent predictor of the outcome. The LOOCV and associated performance evaluation were executed using Python and various libraries, including scikit-learn, statsmodels and seaborn, to support the analysis and generate high-resolution plots for visualisation.

A post-hoc power analysis was conducted. The prospective cohort’s size was constrained by the technically complex experimental setup and patient burden resulting from the associated periods of DBS inactivation. Other studies using motion capture to analyse DBS effects have reported similar sample sizes between 5 and 11 patients in the target groups^6,13,14,61,72^. We approximated the achieved power for our study’s main objective (correlation between gold standard and computer vision tremor amplitudes), post-hoc. Given the observed correlation strength of 0.72–0.89 and an α-error probability of .05, this study achieved a power (1-β) of 0.69 – 0.99 in our sample of 8 participants.

The normality of datasets was examined using the Shapiro-Wilk test and additional inspection of quartile (“Q-Q”) plots to inform the appropriate display of data distributions and the selection of subsequent contrast tests. In case of a significant deviation of the (log-)normality assumption, non-parametric tests, i.e., Wilcoxon rank-sum test and matched rank biserial correlation were used. Linear relationships were examined using Pearson or Spearman’s rank correlations. When appropriate, outliers were removed using the robust regression and outlier removal (ROUT) method with a balanced coefficient of Q = 1%^73^. The significance level was set at *p* < 0.05.

Statistical computations were conducted using Python 3, JAMOVI Version 2.2.5^74^, R Studio^75^ and GraphPad Prism Version 9 (GraphPad Software. GraphPad Prism).

### Reporting summary

Further information on research design is available in the Nature Research Reporting Summary linked to this article.

### Supplementary information

Supplementary Materials

Reporting Summary

Patient consent to disclose main figure

Patient consent to disclose supplementary figure

## Data Availability

Non-identifiable patient data are available upon request to the corresponding author.

## Code availability

Mediapipe hands model is openly available at https://developers.google.com/mediapipe. DeepLabCut code is openly available at https://github.com/DeepLabCut/DeepLabCut. The tremor analysis pipeline is available through github: https://github.com/peach-lucien/PoET. The tremor-specific DLC-RCNN model is available at 10.7910/DVN/CRJRJF.
