# Supplementary material for: Validation and application of computer vision algorithms for video-based tremor analysis
Source: NPJ Digit Med. 2024 Jun 21;7:165. doi: 10.1038/s41746-024-01153-1 (PMC11192937; doi:10.1038/s41746-024-01153-1)
Supplement: Supplementary file 1 — Supplementary Materials [file 41746_2024_1153_MOESM1_ESM.pdf]

Validation and application of computer vision algorithms for video-based tremor analysis

Maximilian U. Friedrich[1,2,3#*], Anna-Julia Roenn[3*], Chiara Palmisano[3], Jane Alty[4], Steffen Paschen[5], Guenther Deuschl[5], Chi Wang Ip [3], Jens Volkmann[3], Muthuraman Muthuraman[3], Robert Peach [3,6*], Martin M. Reich[3*#]

Supplementary figures

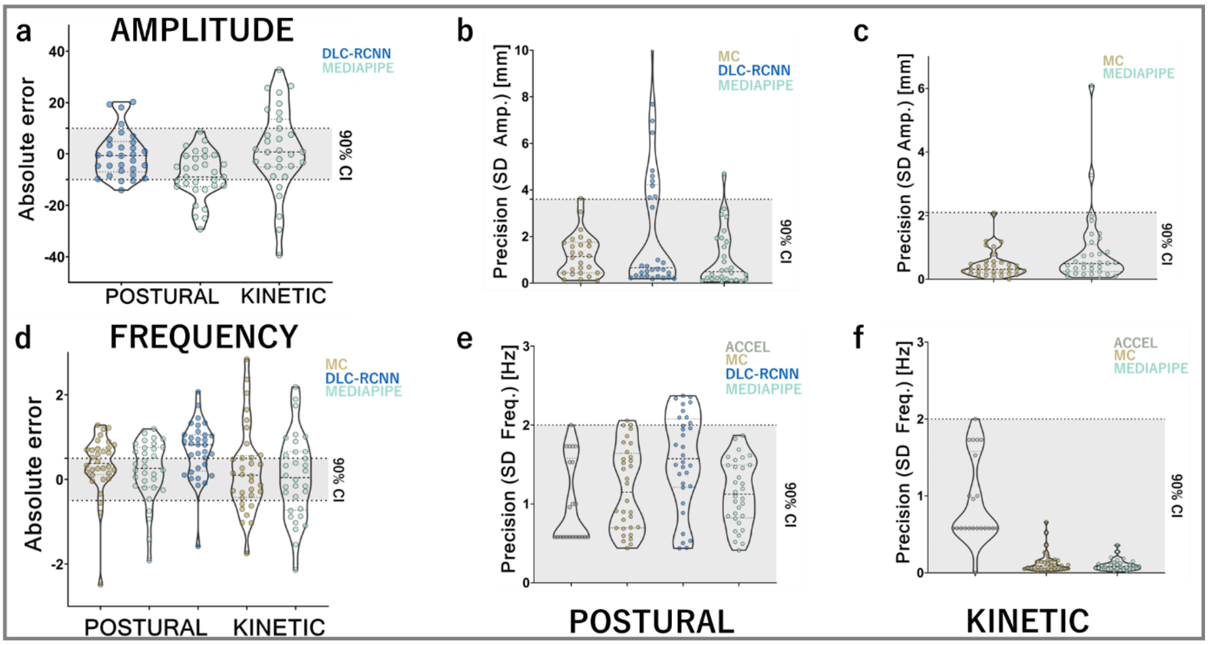

Supplementary Figure 1. **Accuracy and precision assessments in the prospective cohort. a.** Within the a priori defined boundaries of 10 mm, both computer vision algorithms' accuracy in amplitude assessments are equivalent to motion capture (DLC-RCNN: upper limit T(31)= -6.43, lower limit T(31)= 11.3, p< .001, Mediapipe: upper limit T(31)= -14.1, lower limit T(31)= 9.36, p< .001, kinetic: upper limit: T(29)= -4.33, p< .001, lower limit T(29)= 2.57, p= .008). **b.** Precision values of postural tremor amplitude measurements of DLC-RCNN and Mediapipe fall within the equivalence boundaries defined by minimal motion capture precision (DLC-RCNN: upper bound T(25)= -13.3, lower bound T(25)= 11.0; Mediapipe: upper bound T(25)= -25.8, lower bound T(25)= 31.5, all p< .001). **c.** Mediapipe's precision of kinetic tremor amplitude measurements falls within the equivalence boundaries of motion capture (upper bound T(31)= -10.5, lower bound T(31)= 7.13, p< .001). **d.** Mediapipe, but not motion capture and DLC-RCNN show equivalent accuracy to accelerometry in measuring postural tremor frequency (MP: upper bound T(31)= -5.47, p< .001, lower bound T(31)= 2.33, p= .013, DLC-RCNN: upper bound T(31)= -9.9, p< .001, lower bound T(31)= -1.54, p= .930; motion capture: upper bound T(31)= -6.69, p< .001, lower bound T(31)= 1.27, p= .011). Mediapipe-derived kinetic tremor frequencies are equivalently accurate (MP: upper bound T(31)= -3.2, p= .002, lower bound T(31)= 2.52, p= .009). This was not the case for motion capture (upper bound T(31)= -4.0, p< .001, lower bound T(31)= 1.53, p= .068). **e.** Within the boundaries derived from the minimal accelerometry precision, motion capture, DLC-RCNN and Mediapipe exhibit equivalent precision in postural tremor frequency measurements (MC: upper bound T(21)= -17.3, lower bound T(21)= 14.83; DLC-RCNN: upper bound T(21)= -18.0, lower bound T(21)= 9.62, MP: upper bound T(21)= -16.4, lower bound T(21)= 14.62, all p< .001). **f.** Motion capture and Mediapipe exhibit equivalent precision in kinetic tremor frequency measurements (motion capture: upper bound T(21)= -1.66, lower bound T(21)= 14.9; Mediapipe: upper bound T(21)= -9.7, lower bound T(21)= 23.1, all p< .001).

POSTURAL | MEAN AMPLITUDES

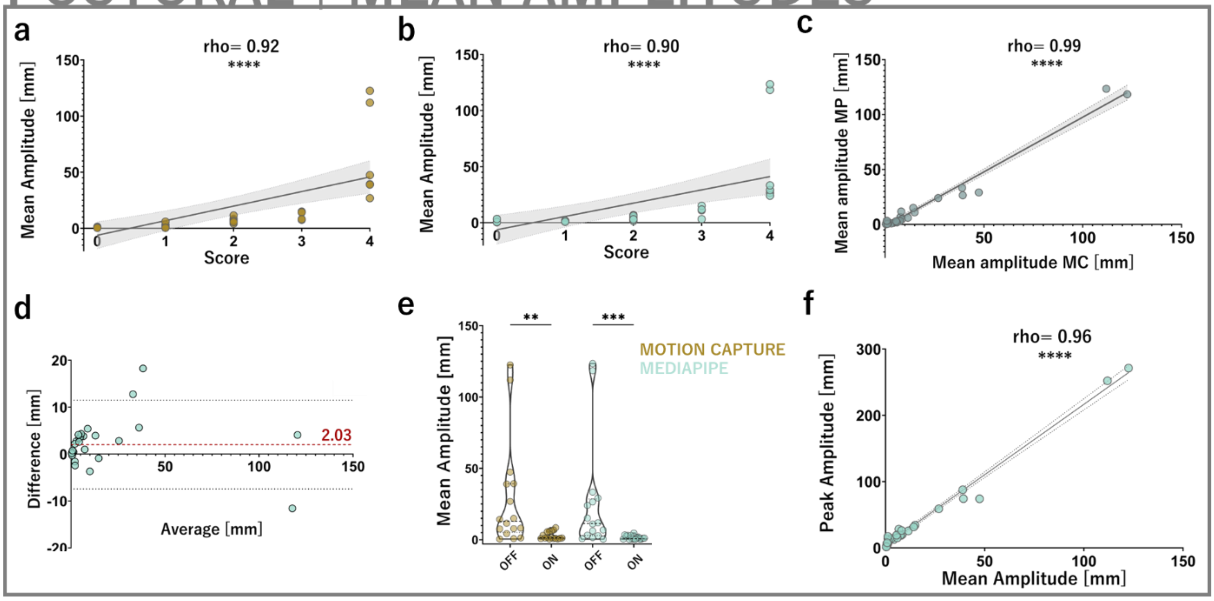

*Supplementary Figure 2. **A-b.*** Repeating **postural** tremor amplitude analyses using *mean* instead of *peak* amplitude measurements yields similarly strong convergent clinical validity and **c.,** intermethodological agreement. **e.** Bland-Altman analysis demonstrates a mean absolute error of 2.03mm of Mediapipe-derived mean postural tremor amplitudes. **e.** Mediapipe-derived mean postural tremor amplitude is responsive to DBS effects (effect size 1.0 Mediapipe, 0.94 motion capture, p< .01). **f.** Mediapipe-derived *peak* and *mean* postural amplitude measurements are strongly correlated.

KINETIC | MEAN AMPLITUDES

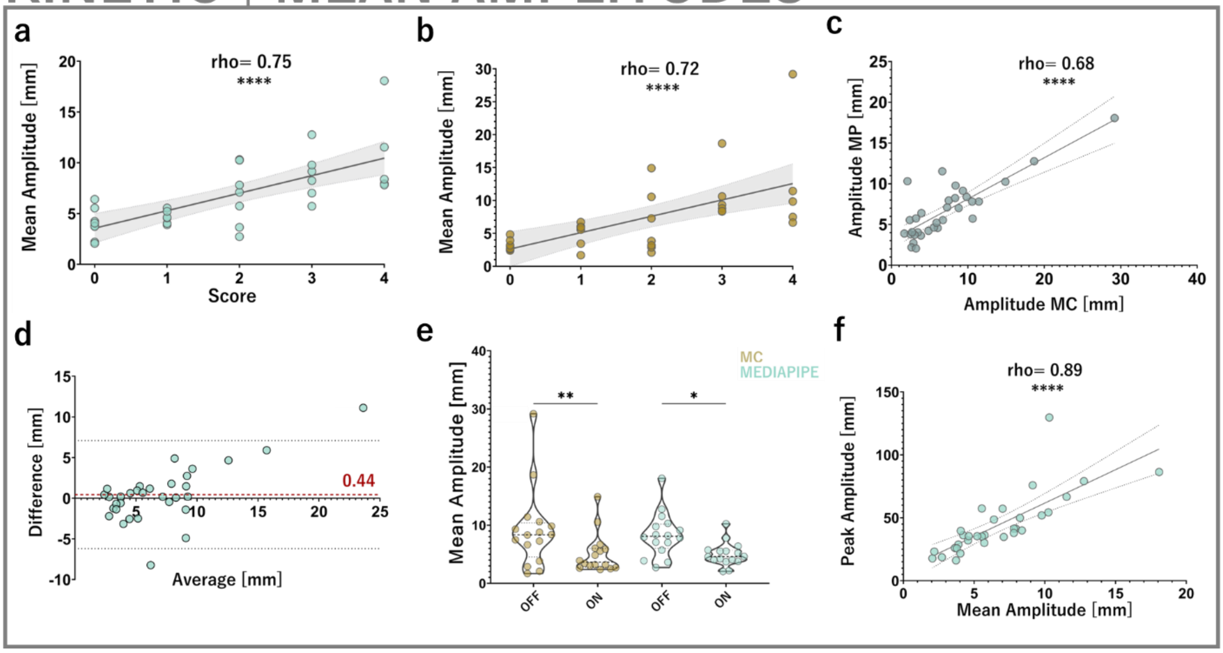

Supplementary Figure 3. **A-b.** Repeating **kinetic** tremor amplitude analyses using *mean* instead of *peak* amplitude measurements yields similar (if not higher) convergent clinical validity (MP: rho= 0.75, MC: rho= 0.72, both p< .001) and **c.**, intermethodological agreement (rho= 0.68, p< .001). **d.** Bland-Altman analysis demonstrates a mean absolute error of 0.44mm of Mediapipe-derived mean kinetic tremor amplitudes. **e.** Mediapipe-derived mean kinetic tremor amplitude is responsive to DBS effects (effect size 0.86 Mediapipe, 0.89 motion capture, p< .05). **f.** Mediapipe-derived peak and mean kinetic amplitude measurements are strongly correlated (0.89, p< .001).

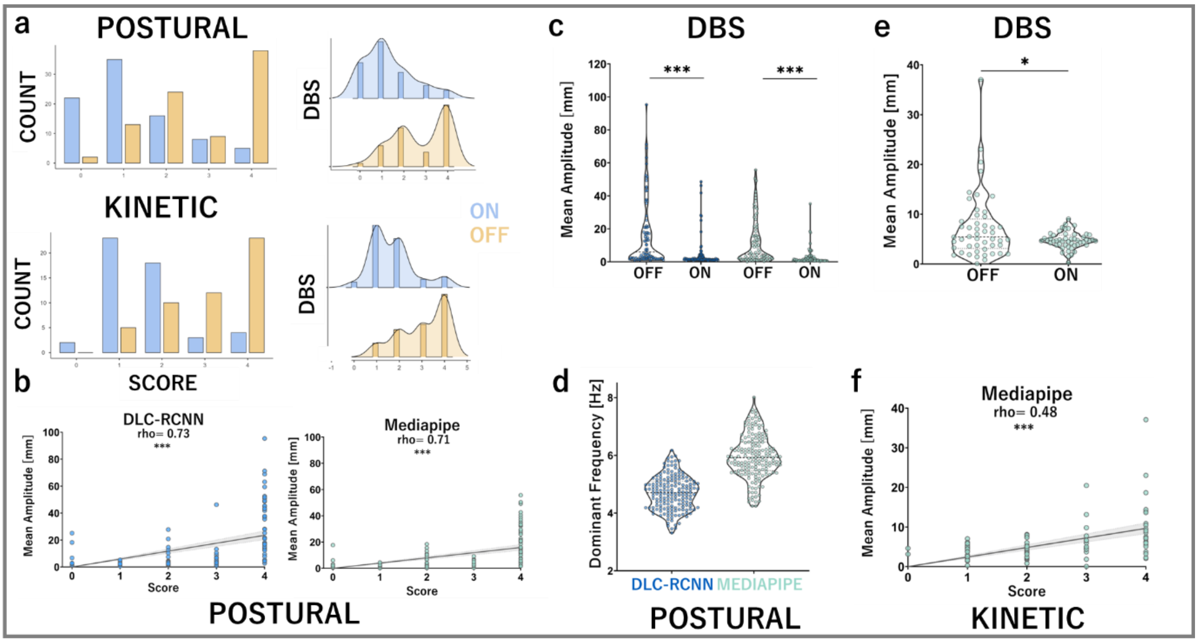

Supplementary Figure 4. **Additional analyses in the retrospective cohort. a.** Tremor score distributions broken down into experimental and treatment conditions. **b.** Mean postural tremor amplitudes are significantly and strongly correlated to clinical scores *(DLC-RCNN: rho= 0.73, p< .001, MP: rho= 0.71, p< .001)*, and **c.** responsive to DBS effect (*(DLC-RCNN: p< .001, r= 0.57 (95% CI [0.45, 0.68], MP: p< .001, r= 0.76 (95% CI [0.43, 0.91]*). **d.** DLC-RCNN-derived tremor frequencies are lower compared to Mediapipe. **e.** Mean kinetic tremor amplitudes are responsive to DBS (*W= -435, p= .03, r= 0.36 (95% CI [0.11, 0.70])* and **f.**, moderately correlated to respective clinical scores (*rho= 0.48, p< .001)*

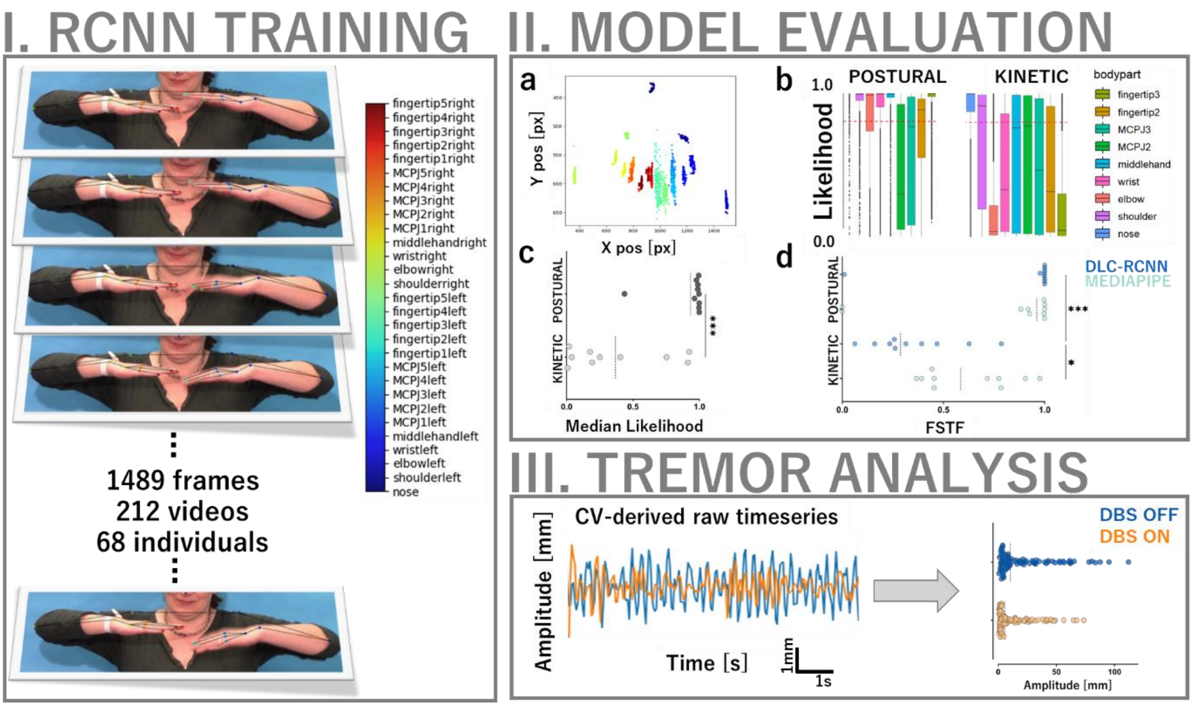

Supplementary Figure 5 - **Tuning a disease-specific CV model.** A residual convolutional neural network, RCNN, is fine-tuned for limb tracking by annotating 1489 frames from 212 videos of 68 individuals with essential tremor across different task and neurostimulation conditions. **a.** Multifaceted model evaluation shows plausible tracking of bilateral keypoints in an aggregated plot, averaging each keypoint's framewise 2D coordinates over time. b. Tracking confidence however varies as a function of anatomical keypoint position such as the hand metacarpophalangeal joints (MCPJ) and task condition, i. e. postural or kinetic tremor assessment (**b-c**). **d.** While both DLC-RCNN and the benchmark hand tracking algorithm, Mediapipe, achieve a similar "fraction of successfully tracked frames" (FSTF, defined as confidence > 0.8) in postural tremor assessments, DLC-RCNN largely fails to track the limbs in the kinetic tremor condition (Friedman *χ2= 16.2, p= .001*, post-hoc test significant in kinetic tremor condition (p= .012). Finally, raw 2D coordinate time series extracted by both computer vision frameworks are transformed into metric tremor amplitudes using a patient- and video-specific calibration procedure based on real-world ground truth dimensions derived from neuroimaging. Informed consent to disclose images was obtained from the patient shown.

Supplementary results

Exploration of peak versus mean amplitude measurements

To ensure our results were independent of the amplitude feature selection, we repeated the main analyses in the validation cohort with either peak or mean amplitudes. In the postural tremor condition, we found a similarly high convergent clinical validity of computer vision and gold standard (rho= 0.90 vs. rho= 0.92, Supplementary Figure 3a-b). Mean amplitude values exhibited high correspondence between methods (rho= 0.99), a mean absolute error of 2.03mm and were highly responsive to the effect of DBS (Supplementary Figure 3c-e). Finally, computer vision-derived mean and peak amplitudes were strongly correlated (rho ≥0.89, Supplementary Figure 3f). Similar results were obtained for kinetic tremor (rho= 0.89, Supplementary Figure 4). Comparable outcomes were observed in the retrospective cohort (Supplementary Figure 5).

Next, we hypothesized that peak amplitudes are less susceptible to intraindividual tremor variability. To explore this, we computed the coefficients of variations (CoV) of each hand's tremor trace as a standardized variability measure (30-90s of recording). We found a nominally lower CoV in the postural than the kinetic paradigm, aligning with the specific task dynamics (postural: median 0.50, IQR 0.29; kinetic: 1.2, IQR 0.3). As expected, the coefficient of variation was more strongly correlated to the mean than peak amplitude measures (postural, mean: rho= -0.3, $p < .001$, peak: rho= -0.21, p= .007; kinetic, mean: rho= -0.42, p= .007, peak: rho= -0.003, p= .97). These findings suggest a considerable variance of tremor amplitudes particularly in the kinetic tremor condition, which aligns with the task dynamics. This variance was found to be more strongly linked to mean than peak amplitudes. Given that peak amplitudes reflect patients' disability more directly and align more closely with the scoring logic, we eventually decided to focus on this feature.

Using the computer vision approach to explore key clinical and demographic characteristics of tremor

In addition to the clinical and technical validation, we also explored how well computer vision-derived tremor features captured established clinical and demographic concepts of essential tremor. First, we focused on the relationship of disease duration and DBS efficacy, which can vary considerable between different movement disorders[20,21]. In line with previous reports[20], we found no significant correlation between disease duration and DBS effect in the prospective cohort, as measured by score differences (postural: rho = 0.48, p = .11; kinetic: rho = 0.48, p= .11) or percentual change of CV-derived tremor amplitudes (postural: rho = -0.27 p= .40; kinetic: rho = -0.31, p= .33). This was also true for the retrospective cohort (scores postural: rho = 0.04, p= .72; kinetic: rho = -0.06, p= .70; CV-derived tremor amplitudes: postural rho = -0.03, p = .80; kinetic: rho = -0.25, r = .085).

Next, we explored the degree of tremor symmetry between limbs, which can vary greatly between essential tremor (more symmetric) and other tremor syndromes such as Parkinson's tremor (more asymmetric)[22]. In line with this notion, we found that tremor amplitudes did not significantly differ between hands (postural tremor scores: W= 204.5, mean difference (MD)= 0, 95% CI [-0.5, 1], kinetic tremor scores: W= 70.0, MD= -0.5 [-1.5, 0.5], CV-derived postural tremor amplitudes: W= 656.0, MD= 0.26mm [-2.7, 3.6], CV-derived kinetic tremor amplitudes: W= 369.0, MD= 5.6mm [0.04, 17.0], all p> .05).

Finally, we explored the effect of adjunct anti-tremor medication (propranolol or primidone) on tremor features during assessments with activated DBS. We split the total sample in two subgroups, one with additional anti-tremor medication (postural n= 15/51, kinetic n= 7/33) and the other without medication. Only the CV-derived median postural, but not kinetic, tremor amplitudes differed between subgroups with vs. without adjunct medication (3.15mm vs. 7.28mm, p< .001). Importantly, there were no significant differences in DBS response between subgroups (postural: 56% vs. 66%, kinetic: 32% vs. 22%, p= .96).

Supplementary references

1. Fasano, A. *et al.* Gait ataxia in essential tremor is differentially modulated by thalamic stimulation. *Brain* **133**, 3635–3648 (2010).
2. Groppa, S. *et al.* Physiological and anatomical decomposition of subthalamic neurostimulation effects in essential tremor. *Brain* **137**, 109–121 (2014).
3. Herzog, J. *et al.* Kinematic analysis of thalamic versus subthalamic neurostimulation in postural and intention tremor. *Brain* **130**, 1608–1625 (2007).
4. Deuschl, G., Bain, P., Brin, M. & Committee, A. H. S. Consensus Statement of the Movement Disorder Society on Tremor. *Movement Disorders* **13**, 2–23 (1998).
5. Fahn, S., Tolosa, E. & Marin, C. Clinical Rating Scale for Tremor. **2**, 271–280 (1988).
6. Friedrich, M. U. *et al.* Smartphone video nystagmography using convolutional neural networks: ConVNG. *J Neurol* (2022) doi:10.1007/s00415-022-11493-1.
7. Seethapathi, N., Wang, S., Saluja, R., Blohm, G. & Kording, K. P. Movement science needs different pose tracking algorithms. Preprint at https://doi.org/10.48550/arXiv.1907.10226 (2019).
8. Williams, S. *et al.* Accuracy of Smartphone Video for Contactless Measurement of Hand Tremor Frequency. *Movement Disorders Clinical Practice* **8**, 69–75 (2021).
9. Stenum, J., Rossi, C. & Roemmich, R. T. Two-dimensional video-based analysis of human gait using pose estimation. *PLOS Computational Biology* **17**, e1008935 (2021).
10. Vissani, M. *et al.* Impaired reach-to-grasp kinematics in parkinsonian patients relates to dopamine-dependent, subthalamic beta bursts. *npj Parkinsons Dis.* **7**, 1–10 (2021).
11. Nath, T. *et al.* Using DeepLabCut for 3D markerless pose estimation across species and behaviors. *Nat Protoc* **14**, 2152–2176 (2019).
12. Haglin, J. M., Jimenez, G. & Eltorai, A. E. M. Artificial neural networks in medicine. *Health Technol.* **9**, 1–6 (2019).

13. Muthuraman, M., Hossen, A., Heute, U., Deuschl, G. & Raethjen, J. A new diagnostic test to distinguish tremulous Parkinson's disease from advanced essential tremor. *Mov Disord* **26**, 1548–1552 (2011).
14. Lauk, M. *et al.* Side-to-side correlation of muscle activity in physiological and pathological human tremors. *Clin Neurophysiol* **110**, 1774–1783 (1999).
15. Anvari, F. & Lakens, D. Using anchor-based methods to determine the smallest effect size of interest. *Journal of Experimental Social Psychology* **96**, 104159 (2021).
16. Lakens, D. Equivalence Tests: A Practical Primer for t Tests, Correlations, and Meta-Analyses. *Social Psychological and Personality Science* **8**, 355–362 (2017).
17. Quantifying Tremor in Essential Tremor Using Inertial Sensors—Validation of an Algorithm. *IEEE J Transl Eng Health Med* **9**, 2700110 (2020).
18. Reich, M. M. *et al.* Progressive gait ataxia following deep brain stimulation for essential tremor: adverse effect or lack of efficacy? *Brain* **139**, 2948–2956 (2016).
19. Fasano, A. *et al.* Lower limb joints kinematics in essential tremor and the effect of thalamic stimulation. *Gait & Posture* **36**, 187–193 (2012).
20. Sandoe, C. *et al.* Predictors of deep brain stimulation outcome in tremor patients. *Brain Stimul* **11**, 592–599 (2018).
21. Isaias, I. U. *et al.* Factors predicting protracted improvement after pallidal DBS for primary dystonia: the role of age and disease duration. *J Neurol* **258**, 1469–1476 (2011).
22. Louis, E. D., Wendt, K. J., Pullman, S. L. & Ford, B. Is Essential Tremor Symmetric?: Observational Data From a Community-Based Study of Essential Tremor. *Archives of Neurology* **55**, 1553–1559 (1998).
