# Supplementary material for: Validation and application of computer vision algorithms for video-based tremor analysis
Source: NPJ Digit Med. 2024 Jun 21;7:165. doi: 10.1038/s41746-024-01153-1 (PMC11192937; doi:10.1038/s41746-024-01153-1)
Supplement: Supplementary file 3 — Patient consent to disclose main figure [file 41746_2024_1153_MOESM3_ESM.pdf]

Universitätsklinikum Würzburg
Klinikum der Bayerischen Julius-Maximilians-Universität

Neurologische Klinik und Poliklinik
Direktor: Prof. Dr. J. Volkmann
Neurochirurgischen Klinik und Poliklinik
Direktor: Prof. Dr. R.-I. Ernestus

Einverständniserklärung – Video- und Fotoaufnahmen

Josef-Schneider-Str. 11, 97080 Würzburg

Patientendaten:

Aufkleber

Albert, Helmut
30.12.1951

Fotoabteilung
der neurologischen und neurochirurgischen Universitätsklinik Würzburg
Tel.: 0931/201-23 675

Hiermit erkläre ich mich damit einverstanden, dass Fotos und/oder Videoaufnahmen von mir zur späteren Verwendung in meiner Behandlung (Verlaufs- und Therapiekontrolle) und zum Meinungsaustausch mit auswärtigen Spezialisten archiviert werden.

Darüber hinaus bin ich einverstanden, dass die Fotos und/oder Videoaufnahmen ggf. verwendet werden dürfen für

- wissenschaftliche Publikationen und wissenschaftliche Vorträge:

  ☒ Ja ☐ Nein

- Fort- und Ausbildungszwecke von Personen, die der ärztlichen Schweigepflicht unterliegen (z.B. Studenten, Ärzte, Pflegepersonal, etc.):

  ☒ Ja ☐ Nein

- Anschauungsmaterial für Personen, die <u>nicht</u> der ärztlichen Schweigepflicht unterliegen (z.B. Vorträge vor Selbsthilfegruppen, Patienten, etc.):

  ☒ Ja ☐ Nein

Eine Veröffentlichung der Fotos und/oder Videoaufnahmen in öffentlichen Medien (Zeitungen, Fernsehen, etc.) bedarf jedoch in jedem Falle einer erneuten Zustimmung durch mich.

Würzburg, 27.7.2021
Ort, Datum

[illegible]
Unterschrift Patient

Diagnose (bitte eintragen!): essentieller Tremor
