# Supplementary material for: Validation and application of computer vision algorithms for video-based tremor analysis
Source: NPJ Digit Med. 2024 Jun 21;7:165. doi: 10.1038/s41746-024-01153-1 (PMC11192937; doi:10.1038/s41746-024-01153-1)
Supplement: Supplementary file 4 — Patient consent to disclose supplementary figure [file 41746_2024_1153_MOESM4_ESM.pdf]

UK
SH

UNIVERSITÄTSKLINIKUM
Schleswig-Holstein

Campus Kiel

Klinik für Neurologie
Neurozentrum
Direktor: Prof. Dr. med. G. Deuschl

EINVERSTÄNDNIS-ERKLÄRUNG für Videoaufnahme

Name: Andreasson

Vorname: Marion

Geburtsdatum: 11.07.1967

Diagnose: ET

Station: N2

Ich erkläre mich damit einverstanden, dass die von mir - meinem Angehörigen - in der Neurologischen Universitätsklinik Kiel hergestellten Videoaufzeichnungen bei der Fortbildung von Fachpersonal und für wissenschaftliche Veröffentlichungen sowie bei Kongressen und in Diskussionen mit anderen Spezialisten vorgeführt bzw. abgedruckt werden.

Kiel, den 11.02.2014

M. Andreasson
(Unterschrift)
